# Mitophagy Transcriptome: Mechanistic Insights into Polyphenol-Mediated Mitophagy

**DOI:** 10.1155/2017/9028435

**Published:** 2017-05-25

**Authors:** Sijie Tan, Esther Wong

**Affiliations:** School of Biological Sciences, Nanyang Technological University, Singapore

## Abstract

Mitochondria are important bioenergetic and signalling hubs critical for myriad cellular functions and homeostasis. Dysfunction in mitochondria is a central theme in aging and diseases. Mitophagy, a process whereby damaged mitochondria are selectively removed by autophagy, plays a key homeostatic role in mitochondrial quality control. Upregulation of mitophagy has shown to mitigate superfluous mitochondrial accumulation and toxicity to safeguard mitochondrial fitness. Hence, mitophagy is a viable target to promote longevity and prevent age-related pathologies. Current challenge in modulating mitophagy for cellular protection involves identification of physiological ways to activate the pathway. Till date, mitochondrial stress and toxins remain the most potent inducers of mitophagy. Polyphenols have recently been demonstrated to protect mitochondrial health by facilitating mitophagy, thus suggesting the exciting prospect of augmenting mitophagy through dietary intake. In this review, we will first discuss the different surveillance mechanisms responsible for the removal of damaged mitochondrial components, followed by highlighting the transcriptional regulatory mechanisms of mitophagy. Finally, we will review the functional connection between polyphenols and mitophagy and provide insight into the underlying mechanisms that potentially govern polyphenol-induced mitophagy.

## 1. Introduction

Mitochondria are energy-generating organelles that synthesize adenosine triphosphate (ATP) to support various cellular activities. Numerous recent studies further advocate an expanded role of the organelle in regulating plethora signalling pathways for cellular survival and homeostasis [1–3]. Mitochondria are also the principal sites of reactive oxygen species (ROS) production inside the cells. Cytosolic ROS need to be tightly regulated to prevent cellular redox imbalance that contributes to the cumulative oxidative damage of macromolecules observed in aging and diseases [4]. Mitochondrial health is a key determinant to the level of ROS produced by the mitochondria. Compromised mitochondrial fitness diminishes cellular bioenergetics, disrupts signalling events, and heightens ROS production [5]. The pivotal roles of mitochondria in various cellular processes highlight the importance of maintaining healthy mitochondrial populations to ensure cellular functions and survival.

Mitophagy plays an instrumental role in influencing mitochondrial health and quality control by eliminating damaged mitochondria in the lysosomes [6–8]. Defects in mitophagy result in accumulation of dysfunctional mitochondria seen in aging and age-related disorders [9–11]. Conversely, upregulation of mitophagy successfully ameliorates mitochondrial dysfunction and cell toxicity in diseases like diabetes mellitus (DM) and Parkinson's disease [12, 13]. Most significantly, enhanced mitophagy activity extends lifespan and healthspan in *Caenorhabditis elegans* (*C. elegans*) and mouse models [14–17]. Currently, mitophagy activity is mainly known to be induced by mitochondrial stress while knowledge of physiological ways to regulate mitophagy lacks behind. A few recent studies indicate that the master transcriptional factors that regulate the expression of autophagy and lysosomal genes can be specifically induced by mitochondrial stress to orchestrate expansion of autophagy-lysosomal fitness to perform mitophagy [18–22]. These transcription factors include forkhead transcription factor (FOXO) and transcription factor EB (TFEB) [18–23], which serve as potential therapeutic targets for modulating mitophagy.

Modulation of dietary intake via the consumption of polyphenol-enriched functional food has been widely researched as a health-promoting measure associated with longevity [24]. Multiple lines of evidence suggest that the beneficial effects of polyphenols, in part, can be attributed to its ability to upregulate mitophagy [25, 26]. Notably, recent studies support a role of polyphenols in influencing the transcriptional regulation of autophagy via the FOXO and TFEB signalling axes to upregulate mitophagy [27–30]. These studies demonstrate that polyphenols modulate mitophagy transcriptome as part of its protective mechanisms to counteract mitochondrial stress [31–33], further strengthening the attractiveness of polyphenols as a therapy for mitochondrial-related pathologies and aging.

In this review, we look at the different surveillance mechanisms involve in the removal of damaged mitochondrial contents, with specific focus on the transcriptional regulation of mitophagy in response to mitochondrial stress. We will also review the functional connection between polyphenols and mitophagy. Based on these reported findings, we propose a mechanistic model by which the intracellular environment senses the administration of polyphenols, to transcriptionally upregulate autophagy and mitophagy genes expression to enhance mitophagy for cellular protection.

## 2. Mitochondrial Quality Control: Different Types of Mitophagy

Besides generating ATP via oxidative phosphorylation to fuel all energy-consuming processes, mitochondria also participate in myriad cellular processes such as ion homeostasis, oxygen sensing, apoptosis, and specification of cell fate in adult and cancer stem cells [2, 3, 34, 35]. Mitochondria are also the prime sites of endogenous ROS production. Mitochondrial health determines the levels of mitochondrial ROS produced. While low amounts of ROS generated by redox competent mitochondria serve important signalling functions, excessive ROS production by dysfunctional mitochondria causes oxidative stress and damage [3, 4]. Safeguarding mitochondrial functions and integrity is, thus, of utmost importance to cell survival.

Mitochondrial homeostasis is complex, and its regulation includes several aspects: mitochondrial dynamics, biogenesis, and the timely removal of worn-out portions [36–41]. Mitochondria undergo continuous fission and fusion events that allow the organelle to alter its shape and size. Such plasticity permits quick adaptation of mitochondrial functions in response to intracellular and extracellular cues [2]. Mitochondrial fission and fusion are also involved in mitochondrial biogenesis and clearance, and the interplay of these processes ensures constant mitochondrial renewal [2]. Deregulation of these processes underlies mitochondrial-related disorders, highlighting the therapeutic prospect of treating human diseases by manipulating mitochondrial biology [42].

Recent insights into mitochondrial quality control via organelle turnover revolutionized our understanding of how a cell vigorously protects itself from dysfunctional mitochondria through multiple defense mechanisms [6–8]. Cells can eliminate different types of damaged mitochondrial contents to cope with varying degrees of mitochondrial stress. During mild mitochondrial stress, such as when mitochondrial proteostasis is perturbed by deranged expression, impaired import, misfolding, or aggregation of mitochondrial proteins, mitochondrial unfolded protein response (UPR^mt^) serves as the first line of mitochondrial quality control [36–38]. UPR^mt^ resolves proteotoxicity in mitochondria by activating a transcriptional response to promote folding, limit import, reduce translation, and enhance degradation of deleterious mitochondrial proteins [36–38]. Mild oxidative stress that inhibits the respiratory chain without causing mitochondrial membrane depolarization leads to the selective incorporation of oxidized mitochondrial proteins into mitochondrial-derived vesicles (MDVs). These MDVs are then delivered to the lysosomes for degradation [43]. This form of mitochondrial component self-eating is independent of autophagy. Instead, the MDVs are engulfed by multivesicular bodies that will subsequently fuse with the lysosomes [8].

**Figure 1:**
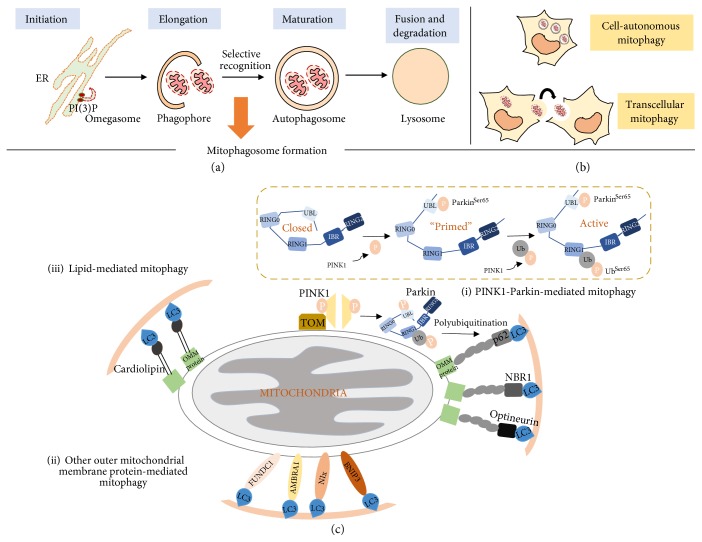
Different mechanisms of mitophagy. (a) Autophagy cascade showing initiation of autophagosome membrane formation from endoplasmic reticulum (omegasome), elongation of the early autophagosome membrane (phagophore), engulfment of mitochondria into the matured autophagosome to form mitophagosome, and final fusion with lysosome for degradation. (b) Mitophagy can occur intracellularly (autonomous) or via a transcellular process where damaged mitochondria are exported to neighbouring cell for degradation by mitophagy. (c) (i) The most well-studied mitophagy pathway is mediated by PINK1 and Parkin. Under mitochondrial stress, PINK1 is stabilized on the outer mitochondrial membrane (OMM) where it associates with the TOM complex. Subsequently, PINK1 undergoes dimerization and autophosphorylation to promote recruitment of Parkin to the OMM for its activation. Under physiological conditions, Parkin adopts a closed confirmation and is kept inactive due to the association between its UBL and RING1 domain. Mitochondrial membrane depolarization causes PINK1 to phosphorylate Parkin at Ser65 which perturbs the UBL-RING1 association. PINK1 mediates a second phosphorylation event on the ubiquitin (Ub) molecule at Ser65 to fully activate the E3 ligase activity of Parkin through phosphorylated Ub^Ser65^ binding. Activated Parkin ubiquitinates OMM proteins via K27 or K63-linked polyubiquitination to serve as recognition tags to recruit cargo adaptor proteins like p62, NBR1, and optineurin for selective targeting of mitochondria into the autophagosome. OMM proteins can also directly bind phosphorylated Ub^Ser65^ to recruit cargo adaptors. (ii) OMM proteins such as BNIP3, NIX, FUNDC1, and AMBRA1 also possess the LIR motif and can interact directly with LC3 to facilitate selective targeting of the mitochondria to the autophagosome. (iii) Lipid moieties on the OMM can alternatively bind LC3 independent of cargo adaptor proteins. For example, phospholipid cardiolipin has shown to translocate from the inner mitochondrial membrane to the OMM to recruit LC3.

In the event of severe oxidative stress leading to global mitochondrial damage due to mitochondrial depolarization, sequestration of individual damaged mitochondria into autophagosomes is activated for targeted disposal via the autophagy-lysosomal pathway [6–8]. This form of selective removal of dysfunctional mitochondria by autophagy is known as mitophagy (*mito*chondria + auto*phagy*), which eliminates damaged mitochondria while preserving the integrity of the remaining healthy mitochondria [44]. Hence, mitophagy represents an important quality control pathway to monitor mitochondrial health and homeostasis [6–8]. Besides degradation of damaged mitochondria in cell autonomous manner, recent studies have reported the discovery of a transcellular mitophagy phenomenon in the mice optic nerve head [45, 46]. Unlike classical mitophagy, damaged mitochondria in the retinal ganglion cells are delivered to the neighbouring astrocytes for degradation by mitophagy (Figure 1()). The transcellular mitochondrial transfer is facilitated by the release of mitochondria-filled axonal vesicles from the retinal ganglion cells into the extracellular spaces for uptake by the astrocytes. Distinct membrane-enclosed evulsions containing mitochondria were seen in astrocytes proximal to the ganglion axonal projections [45]. Whether this type of mitophagy occurs in other neuronal cell types remains to be elucidated [46]. Nonetheless, the varied sophisticated measures put in place by the cell to maintain mitochondrial quality control highlight the significance of timely and accurate destruction of toxic mitochondrial portions. Mitophagy is currently the best characterized form of mitochondrial quality control. Research into the discovery and understanding of the components and mechanisms of UPR^mt^ and MDVs, although intriguing, are still in its infancy. Hence, in this review, we will focus on mitophagy with regard to polyphenol modulation of mitochondrial quality control.

Mitophagy declines with age, and the impairment in mitochondrial clearance is associated with several human pathologies. This includes age-associated disorders such as cancer, metabolic syndrome, and neurodegeneration [9, 47–49]. On the other hand, upregulation of mitophagy mitigates disease progression and protects against diseases. This has been demonstrated in DM. Accumulation of advanced glycation end products (AGEs) due to chronic hyperglycaemia induces glycoxidative stress in DM, leading to massive mitochondrial dysfunction. Mitophagy is important in protecting cells from mitochondrial toxicity in DM condition [12]. Indeed, mitophagy induction in diabetic platelets protects it against oxidative stress-induced mitochondrial damage and apoptosis, thereby reducing thrombotic injuries in DM [50]. Mitophagy therefore displays therapeutic potential for treating human diseases. Elucidation of the mechanisms governing mitophagy holds a promise for the development of novel pharmacological interventions to delay aging and to prevent age-related pathologies. Current challenges in modulating mitophagy for cellular protection include delineating the different variants of mitophagy and identification of more physiological ways to activate mitophagy or individual mitophagy variants. Ironically, mitochondrial toxins such as CCCP, a strong mitochondrial uncoupler, remain the most effective inducer of mitophagy but with accompanying grave mitochondrial damage. There is a clear need to search for drugs or natural compounds that activate mitophagy without incurring such undesirable side effects. Here, we will review the current understandings on natural polyphenolic modulators of mitophagy and their associated benefits and mechanisms.

### 2.1. Formation of Mitophagosome: When Mitochondria Meet Autophagy

Autophagy is a catabolic process where unwanted or damaged intracellular constituents are engulfed in autophagosomes for delivery to lysosomes for degradation (Figure 1). The degradation pathway consists of four stages: (1) initiation, (2) elongation of the autophagosomal membrane, (3) maturation of the autophagosome, and (4) fusion of the autophagosome with the lysosome [51]. A set of conserved proteins encoded by the autophagy-related (*atg*) genes control the different stages of the autophagy cascade. In yeast, more than 30 *atg* genes have been characterized thus far and the mammalian orthologs have also been subsequently identified [52]. The strong evolutionary conservation of the *atg* genes across lower and higher eukaryotes highlights the critical role of autophagy in the maintenance of cellular homeostasis and survival.

Under physiological conditions, basal autophagy is important for constitutive turnover of proteins and organelles for quality control critical to sustain cellular activities. The functional importance of basal autophagy is highlighted in several studies. For example, brain-specific ablation of the autophagy pathway via Atg5 and Atg7 knockouts in mice caused an accumulation of ubiquitinated protein inclusions accompanied by neurodegenerative deficits [53, 54]. Liver-specific inhibition of autophagy led to the development of multiple liver tumors [55], and systemic inhibition of autophagy in mice resulted in neonatal lethality [56]. These studies highlight the importance of basal autophagy as a protective mechanism to prevent accumulation of damaged or redundant cellular constituents and, consequently, the development of diseases in healthy organisms.

In addition to basal autophagy, autophagy can be upregulated as an adaptive response to cope with cellular stress as seen in the case for mitophagy. Stress-inducible autophagy is now increasingly recognized for its selectivity, wherein specific substrates damaged by a particular stressor are targeted for lysosomal degradation while the remaining cellular milieu is preserved [57]. In mitophagy, the selective recognition and precise loading of dysfunctional mitochondria into the autophagosomes to form mitophagosomes underlies the targeted removal of damaged mitochondria (Figure 1). The selectivity of mitophagosomes is facilitated by specialized autophagy receptors known as cargo adaptor proteins. These adaptors contain a microtubule light chain 3- (LC3-) interacting region (LIR) motif that binds LC3 in the autophagosomal membrane, thereby linking the substrate to the autophagosome. Till date, several cargo adaptors have been identified for mitophagy and this underscores the different mechanisms of mitophagosome formation which we briefly discuss below.

Prior to mitophagy induction, mitochondria undergo fission (or fragmentation) to sieve out the damaged mitochondria from the healthy mitochondrial network for efficient targeting of the former for degradation [58]. The mitochondrial dynamics is regulated by members of the guanosine-5′-triphosphate (GTP)ase family: (1) dynamin-1-like protein (Drp1), which is a fission-promoting protein and (2) mitofusins 1 and 2, which are fusion-promoting proteins. Drp1 mediates fission by forming a multimeric complex around the mitochondria tubule to induce membrane scission and mitochondria excision [59]. Conversely, mitofusins mediate fusion via dimerization with the adjacent mitofusins on the neighbouring mitochondria to promote membrane tethering between mitochondria [60]. Induction of mitophagy promotes ubiquitination and degradation of mitofusins to favour mitochondrial fragmentation for efficient autophagy targeting [61].

### 2.2. Different Facets of Mitophagosome Formation

#### 2.2.1. PINK1-Parkin-Mediated Mitophagy

The most well-studied mitophagy pathway is mediated by PTEN-induced putative kinase 1 (PINK1) and E3 ligase Parkin (Figure 1(c), (i)). Under physiological conditions, PINK1 is kept inactive in the mitochondrial matrix via cleavage by mitochondrial processing peptidase (MPP) and presenilin-associated rhomboid-like (PARL) protease [62–64]. On the other hand, mitochondrial stress induced by membrane depolarization stabilizes and activates PINK1 on the outer mitochondrial membrane, where it promotes recruitment and activation of Parkin [65, 66].

PINK1-mediated activation of Parkin is orchestrated by an interplay between phosphorylation and ubiquitination. Under physiological conditions, Parkin adopts a closed confirmation and is kept inactive due to the association between the N-terminal ubiquitin-like (UBL) and the RING1 domain (Figure 1()(c), (i)). Mitochondrial membrane depolarization induces Parkin phosphorylation at Ser65 by PINK1, promoting a conformational change and partially relieving the autoinhibition on Parkin [67, 68]. A second phosphorylation event, which involves phosphorylation of the ubiquitin molecule also at Ser65 by PINK1, is an important event for full activation of Parkin. The phosphorylated ubiquitin binds to the UBL domain of Parkin to fully activate the E3 ligase activity [69, 70]. Activated Parkin polyubiquitinates the outer mitochondrial membrane proteins, where the ubiquitination serves as recognition tag for cargo adaptor proteins to initiate mitophagy. In addition, the PINK1-Parkin complex can also directly interact with Beclin-1 in the class III phosphoinositide 3-kinase (PI3K) complex, or indirectly with the PI3K complex via AMBRA1, to initiate autophagosome membrane biogenesis around the damaged organelle [71, 72].

#### 2.2.2. Other Mitochondrial Membrane Proteins and Lipid-Mediated Mitophagy

Apart from the PINK1-Parkin mechanism, mitophagy can alternatively be initiated by other proteins and lipids on the outer mitochondrial membrane (Figure 1()(c), (ii, iii)). BCL2/adenovirus E1B 19 kDa protein-interacting protein 3 (BNIP3), NIP3-like protein X (NIX), and FUN14 domain containing 1 (FUNDC1) are outer mitochondrial membrane proteins that harbour the LIR motif. The outer mitochondrial membrane can, therefore, also directly interact with the autophagosome during mitophagy. Additionally, AMBRA1 participates in both Parkin-dependent and independent mitophagy [73]. In Parkin-independent mitophagy, AMBRA1 binds LC3 directly through its LIR motif. Lipid moieties on mitochondrial proteins similarly can interact with the autophagosome. For example, the inner mitochondrial membrane phospholipid cardiolipin translocates to the outer mitochondrial membrane under mitochondrial stress to interact with LC3 [74, 75]. Ceramide, a sphingolipid on the mitochondrial membrane, has also been shown to interact with LC3 [76].

## 3. Transcriptional Regulation of Mitophagy

**Figure 2:**
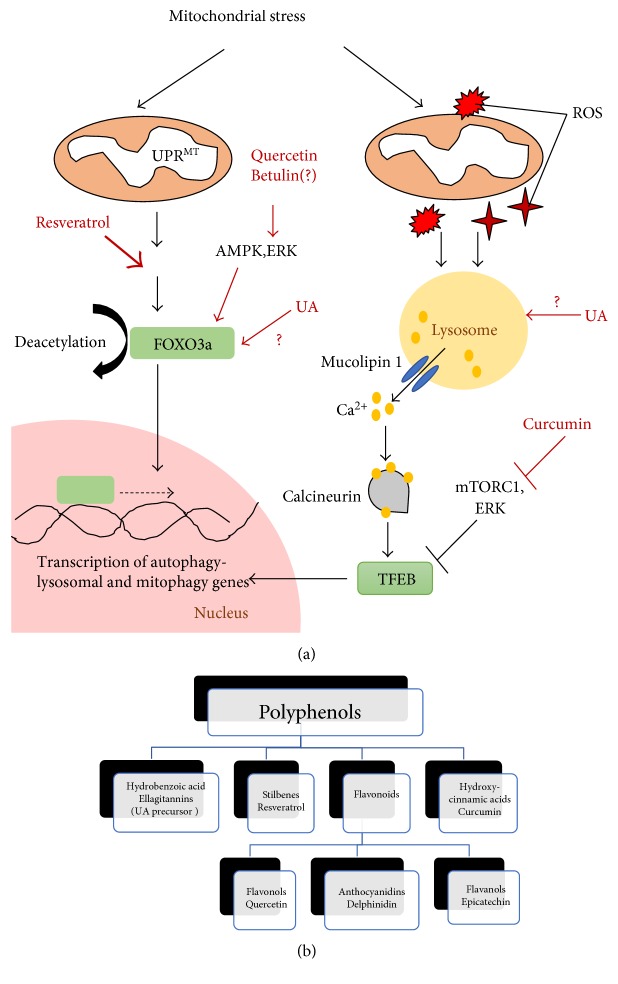
Regulation of FOXO3a and TFEB transcriptional control of mitophagy by polyphenols. (a) Mitochondrial stress activates mitochondrial unfolded protein responses (UPR^MT^) to upregulate expression of SIRT3. SIRT3 deacetylates transcription factor FOXO3a to promote its nuclear translocation. Resveratrol, betulin, and quercetin potentially influence FOXO3a activation via SIRT3 dependent or independent pathways. Mitochondrial stress also generates reactive oxygen species (ROS) to activate the lysosomal Ca^2+^ signalling and calcineurin to dephosphorylate TFEB and promote its nuclear translocation. Curcumin activates TFEB via mTORC1 inhibition. Urolithin A (UA) may modulate mitophagy transcriptome via regulating FOXO3a or TFEB activity. Once in the nucleus, FOXO3a and TFEB initiate transcription of autophagy-lysosomal and mitophagy genes to facilitate mitochondrial clearance. (b) Classification of polyphenols.

Studies have begun to show that transcriptional mechanisms play a pivotal role in modulating autophagy. These transcription factors are often activated in response to lowered nutrient or energy status in order to upregulate expression of autophagy and lysosomal genes to expedite the recycling and generation of amino acids, lipids, and ATP from degraded cellular components. FOXO and more recently, TFEB, are transcription factors that have been intensively studied and established to upregulate autophagy and lysosomal biogenesis under starvation conditions [19, 23, 77, 78]. Activation of FOXO and TFEB transcriptional activities has been linked to beneficial autophagy associated with lifespan extensions in model organisms [79, 80]. Transcriptional upregulation of autophagic flux induced by starvation also enhances protein-organelle quality control where mitochondria may be degraded as part of the autophagic cargoes. It remains unclear whether selection factors are involved in mitochondrial degradation by starvation-induced autophagy [8]. Importantly, recent studies have revealed that FOXO and TFEB specifically response to mitochondrial stress to induce mitophagy by upregulating autophagy and several mitophagy genes. Many lines of emerging evidence suggest that stress-induced transcriptional upregulation of mitophagy has its own unique signalling signature (discussed below) (Figure 2()). Thus, targeting these mitophagy-specific transcriptional signalling pathways serves as an avenue for preferentially inducing mitophagy. The global effects of transcriptional regulation may offer an overarching advantage over interventions aimed at augmenting the formation of specific types of mitophagosomes (Figure 1()). Furthermore, these transcription regulators also coregulate mitochondrial biogenesis in addition to mitochondrial turnover, hence allowing coordinated enhancement of mitochondrial proliferation and degradation to better triage mitochondrial homeostasis [19, 40, 41].

### 3.1. FOXO3a Signalling

FOXO is a family of transcription factors characterized by a conserved DNA-binding domain termed the “forkhead box.” In human, the FOXO family contains four members, namely, FOXO1, FOXO3/3a, FOXO4, and FOXO6 [27]. All 4 FOXO members are expressed in skeletal muscle cells and are implicated in transcriptional activation of genes involved in protein degradation (proteasome and autophagy), glycolysis, lipophagy (selective autophagic degradation of lipid droplets), and mitochondrial respiration for skeletal muscle homeostasis [81]. FOXO is activated by AMP-activated protein kinase (AMPK) and nicotinamide adenine dinucleotide- (NAD^+^-) dependent deacetylase sirtuin-1 (SIRT1) signalling pathways to upregulate autophagy in response to nutrient and energy cues. Low ATP levels activate AMPK to directly induce FOXO nuclear localization and autophagy upregulation [82, 83]. In contrast, a low nutrient supply increases the level of NAD^+^ leading to SIRT1 activation. Activated SIRT1 thereafter deacetylates FOXO to promote its nuclear translocation and transcription activity [84]. Amongst the 4 members, FOXO3 is most frequently associated with autophagy induction. FOXO3 controls the expression of genes involved in autophagosome biogenesis [81, 85–87].

Two recent studies delineate a role of FOXO3a in modulating mitophagy. In the first study, mitochondrial proteotoxic stress activates UPR^mt^ and elevates sirtuin-3 (SIRT3) expression (Figure 2(a)). SIRT3 is another member of the sirtuin family that is localized to the mitochondria and plays predominant roles in mitochondrial processes. A study found that increased SIRT3 levels lead to FOXO3a deacetylation and activation [88]. Active FOXO3a induces the transcription of genes involved in mitophagy, including *lc3*, *atg9*, and *bnip3l/nix* [88]. Notably, the study observed that only the fragmented mitochondria were engulfed by autophagosomes whereas the remaining mitochondrial network was unaffected [88]. This observation is consistent with the notion of mitophagy, where only the damaged mitochondria are targeted for autophagic clearance. It was also observed that Parkin expression levels remained unchanged, suggesting that SIRT3-FOXO3a-mediated mitophagy is independent of Parkin [88]. It appears that the Parkin-independent mitophagy induced by SIRT3-FOXO3a is a peculiar response to mitochondrial UPR. In contrast, another study reported that SIRT3-FOXO3a signalling upregulated Parkin expression to mediate enhanced mitophagy to protect against diabetic cardiomyopathy in mice [89]. This shows that SIRT3-FOXO3a activation can induce different mitophagy mechanisms, possibly determined by the type of mitochondrial stress.

### 3.2. TFEB Signalling

TFEB is the first member of the microphthalmia family of basic helix-loop-helix-leucine-zipper (bHLH-Zip) transcription factors (MiTF) identified to be a master regulator of autophagy-lysosomal genes [90]. TFEB binds to the coordinated lysosomal expression and regulation (CLEAR) motif, a 10-base E-box-like palindromic sequence found in the promoters of autophagy and lysosomal genes, to activate their transcription [91]. Thus far, TFEB is mainly activated by cellular stressors such as starvation and ROS production [20, 92]. ROS regulation of TFEB activity serves as an important route for cells to detect mitochondrial malfunction in order to upregulate transcription of autophagy-lysosomal genes to enhance mitophagy and suppress oxidative stress [20].

In TFEB-induced autophagy, the lysosome acts as a signalling hub that senses changes in amino acid levels in the lysosomal lumen or intracellular ROS levels to regulate TFEB activity. Lysosome regulates TFEB phosphorylation and activation status through 3 signalling cascades: (1) mammalian target of rapamycin complex 1 (mTORC1), (2) extracellular signal-regulated kinase 2 (ERK2), and (3) lysosomal Ca^2+^-activated calcineurin [93–95]. For nutrient regulation of TFEB, high level of amino acids in the lysosomal lumen induces conformational changes in vacuolar-type H^+^-ATPase proton pump (v-ATPase) to stabilize Ragulator complex at the lysosomal surface to recruit and activate mTORC1. Active lysosomal mTORC1 in turn recruits and phosphorylates TFEB at Ser142 and Ser211 to sequester TFEB in the cytosol and render it inactive [93–95]. In addition to mTORC1, Ragulator also promotes translocation of ERK2 towards the lysosome vicinity under amino acid-rich conditions to promote TFEB Ser142 phosphorylation [96]. This mechanism provides another regulatory route to inhibit TFEB activity. Conversely, under amino acid deprivation, Ragulator and mTORC1 are not recruited to the lysosomal surface, which relieves TFEB suppression. Nutrient regulation of TFEB activity is also governed by dephosphorylation of TFEB. Recently, it has been shown that the lysosome responds to starvation by facilitating Ca^2+^ release from the lysosomal lumen through the lysosomal Ca^2+^ channel mucolipin 1 (Figure 2(a)). The localized lysosomal Ca^2+^ release activates calcineurin, a phosphatase that dephosphorylates TFEB to promote TFEB nuclear shuffling and transcription activity [97].

Besides being an effector of the lysosomal nutrient sensing pathway to adapt cell metabolism, TFEB also responds to other cellular stressors to orchestrate plethora homeostatic responses [92]. A recent example is TFEB activation by mitochondrial stress to upregulate autophagy-lysosomal transcriptome for specific removal of dysfunctional mitochondria (Figure 2(a)). The first evidence demonstrating TFEB as a transcriptional regulator of mitophagy came from the observation that mitochondrial depolarizing agents, oligomycin and antimycin A, induced TFEB dephosphorylation and nuclear translocation [98]. Unlike starvation, mTORC1 inactivation is dispensable in TFEB activation by mitochondrial stress, which instead is dependent on Parkin activity [98]. E3 ligase Parkin promotes TFEB-induced mitophagy by degrading Parkin interacting substrate (PARIS), a transcriptional repressor of proliferator-activated receptor-gamma coactivator-1*α* (PGC-1*α*) [99]. PGC-1*α* has recently shown to regulate TFEB expression in addition to mitochondrial biogenesis and energy metabolism. Parkin therefore relieves PARIS inhibition on PGC-1*α* to drive PGC-1*α*-mediated TFEB expression for mitophagy [99]. Perturbation of Parkin activity in Q311X Parkin mutant and sporadic Parkinson's disease mouse models led to an increase in PARIS levels that coincided with disrupted PGC-1*α*-TFEB signalling [13]. Disruption of Parkin intricate control of PARIS level resulted in mitochondrial impairment and degeneration of dopaminergic neuronal cells in these mouse models, which were successfully reversed via upregulation of PGC-1*α*-TFEB signalling [13]. In turn, TFEB reciprocally regulates PGC-1*α* expression to enhance compensatory mitochondrial biogenesis to replenish the mitochondrial pool removed by mitophagy [18]. Therefore, TFEB not only senses the need for increasing autophagy-lysosomal activity in order to degrade damaged mitochondria, but also coordinates the replacement of mitochondria through PGC-1*α*-mediated synthesis of new mitochondria. TFEB hence acts as an integrative node linking mitochondrial quality control by mitophagy to mitochondrial biogenesis to maintain mitochondrial homeostasis.

The question of how the lysosome senses mitochondrial dysfunction to activate TFEB signalling independent of mTORC1 remained elusive until the recent identification of a ROS-lysosome-TFEB signalling mechanism [20]. In this study, ROS production caused by CCCP-induced mitochondrial stress increases Ca^2+^ efflux from lysosome via mucolipin 1 (Figure 2(a)). Addition of reducing or antioxidant reagents prevented activation of mucolipin 1, demonstrating that ROS induces lysosomal Ca^2+^ release. The localized Ca^2+^ surge in the cytosol activates calcineurin-dependent dephosphorylation of TFEB to release TFEB for nuclear shuffling and upregulates autophagosome and lysosome biogenesis to increase the cellular capacity for mitophagy. This study hence demonstrates that ROS may function as protective signalling molecules to upregulate adaptive cellular responses to combat oxidative stress.

## 4. Polyphenols and Mitophagy

Traditionally characterized as secondary metabolites for protection against ROS insults in plants [100], polyphenols are now intensively studied for their health-promoting properties [25]. Indeed, polyphenol-enriched diet can protect against neurodegeneration to favour healthy aging [25]. Mitochondria are a major cellular target of polyphenols. Many polyphenols demonstrate positive effects on mitochondrial biogenesis, integrity, and respiratory capacity [101]. For example, resveratrol has been shown to ameliorate mitochondrial bioenergetics and biogenic impairments in neuronal progenitor cells of the Down syndrome mice model. Resveratrol mitigates by activating mitochondrial biogenesis via PGC-1*α*-SIRT1-AMPK signalling and restoring mitochondrial oxidative phosphorylation capabilities [102]. Low doses of resveratrol also protect against respiration dysfunction induced by mitochondrial mutations in patient-derived fibroblast cells [103]. In another example, rosmarinic acid was reported to attenuate insulin resistance in rat skeletal muscle via enhancing mitochondrial proliferation through increasing mitochondrial synthesis factors such as PGC-1*α*, SIRT1, and transcription factor A mitochondria (TFAM) [104]. Epicatechin, another polyphenol highly enriched in cocoa, was similarly shown to increase the expression of key mitochondrial respiratory and biogenesis factors, including PGC-1*α*, TFAM, and SIRT1, to improve mitochondrial respiratory function in skeletal muscle and myocardial cells [105–113]. Alma, a plant found in traditional Indian medicine, protects against oxidative stress in skeletal muscle cells by upregulating mitochondrial biogenesis and respiration via AMPK activation [114].

While many studies have looked at the influence of polyphenols on mitochondrial synthesis and functions, few have explored the effects of these natural compounds on mitophagy. Although polyphenols have been reported to induce autophagy [25], degradation of mitochondria in these cases is often a consequence of global autophagy upregulation for energy production rather than due to selective mitochondrial clearance. It is only recently that evidence supporting a role of polyphenols in specific transcriptional regulation of mitophagy has been reported. Based on these findings, we propose a mechanistic model on how the general classes of polyphenols (Figure 2(b)) could transcriptionally induce mitophagy to protect against mitochondrial stress.

### 4.1. Polyphenols Enhance FOXO3a Activation to Mediate Mitophagy

Recently, stilbenes (resveratrol) and flavonols (quercetin) have been shown to alter mitophagy transcriptome via FOXO3a signalling to potentiate Parkin-PINK1 mitophagy in cardiac and hepatic cells. These natural compounds upregulate mitophagy, in part, by enhancing the gene expressions of Parkin and PINK1 under myocardial infarction and liver injury (see below).

Resveratrol, a trans-3,4′,5-trihydroxystilbene enriched in grapes and berries, along with its modified form Longevinex, was shown to induce mitophagy to attenuate myocardial infarction in rats subjected to ischemic reperfusion (I/R) injury [27]. In the study, resveratrol and its mimetic induced the acetylation of SIRT3 to activate the downstream effector FOXO3a. Enhanced PINK1 and Parkin localization to the mitochondria were observed in the injured cardiac cells [27]. It is unknown whether FOXO3a activation exerts a direct effect on the transcriptional upregulation of these mitophagy factors. However, PINK1 has been shown to be a downstream target of FOXO3a [115], thus suggesting the possibility that resveratrol and Longevinex may induce PINK1 expression via FOXO3a activation to subsequently facilitate Parkin recruitment to the mitochondria in the infarction area. Enhanced mitochondrial fission was also observed in the infarction area, which was postulated to mediate efficient mitophagy of the fragmented mitochondria [27]. Indeed, the interdependence between mitochondrial fission and Parkin-mediated mitophagy to maintain mitochondria quality has been reported in the hearts of mice undergoing cardiac ischemia [116]. Disruption of mitochondrial fission through Drp1 ablation results in failure to separate the damaged mitochondria from the healthy network leading to perturbed mitophagy and mitochondrial homeostasis [116]. Taken together, resveratrol and Longevinex potentiate mitophagy in cardiac cells by promoting efficient PINK1-Parkin-mediated mitophagy via influencing two factors: (1) enhancing mitochondrial fragmentation and (2) potentially inducing expression of PINK1 in a FOXO3a-dependent manner.

Similar to resveratrol, quercetin was also reported to activate FOXO3a-mediated mitophagy. Quercetin is a flavonoid found enriched in many fruits, vegetables, and grains and has earlier been shown to alleviate mitochondrial oxidative stress via its antioxidant properties in ethanol-induced dyslipidemia [117]. In a recent study, the phenolic compound was shown to protect against mitochondrial damage in ethanol-induced liver injury through activation of mitophagy [28]. Ethanol feeding led to mitochondrial impairment in the mouse liver, characterized by degenerative changes in mitochondrial ultrastructure and membrane potential and fluidity. In addition, repression of Parkin expression and accumulation of partially sequestered mitochondria by the autophagosome were also observed, suggesting inefficient mitophagy during ethanol exposure. Administration of quercetin attenuated the pathological mitochondrial changes and restored mitophagy by activating FOXO3a, unlike resveratrol, in an AMPK- and ERK2-dependent pathway. This was accompanied by reversion of Parkin transcriptional inhibition, enhanced lysosome biogenesis, and fusion with mitophagosomes [28]. Another polyphenol, betulin, was also reported to alleviate ethanol-induced alcoholic liver injury via the SIRT1-AMPK signalling pathway to enhance lipophagy [118]. Whether betulin can concomitantly upregulate mitophagy to attenuate mitochondrial oxidative stress under alcohol-induced hepatotoxicity remains to be elucidated. Its potential to activate AMPK, however, suggests that betulin may similarly be able to activate FOXO3a-dependent mitophagy for hepatic protection.

The role of AMPK signalling as an interface for mitophagy was also observed in anthocyanin delphinidin-3-glucoside- (D3G-) mediated cytoprotection against oxidized low-density lipoprotein (oxLDL) toxicity during vascular endothelial cell injury [106]. D3G-mediated AMPK activation increases NAD^+^ levels to enhance SIRT1 activity which in turn upregulate mitophagy to prevent mitochondria dysfunction in oxLDL injured endothelial cells [119]. The mechanisms underlying D3G-driven SIRT1-mediated mitophagy currently remain unclear. It is possible that FOXO3a may underscore the link between SIRT1 activation and enhanced mitophagy for D3G effects. Alternatively, SIRT1 may directly mediate deacetylation and activation of key autophagic proteins such as Atg5, Atg7, and Atg8 to induce autophagy for mitochondrial removal [120].

### 4.2. Polyphenols and TFEB Signalling in Mitophagy

Since TFEB is regulated by mTORC1 activity, polyphenols that inhibit mTORC1 may be a viable activator of TFEB. However, it remains mostly unknown whether polyphenols that inhibit mTORC1 also influence TFEB signalling and mitophagy. Curcumin, a diferuloylmethane and component of the turmeric plant, is currently the only polyphenol reported to regulate TFEB activity by inhibiting the AKT-mTORC1 signalling pathway [30]. A curcumin analog C1 that possesses better cellular uptake and a longer half-life has been shown to induce TFEB signalling in vitro and in vivo via distinct TFEB activation mechanisms. Unlike curcumin, TFEB activation by C1 is independent of mTORC1 and dephosphorylation events. Instead, C1 binds directly to TFEB at the N-terminal to alter the conformation of TFEB in order to expose its nuclear localization signal to facilitate nuclear translocation [121]. However, whether mitophagy activation is a downstream effector of curcumin-induced TFEB activation has yet to be addressed and warrants future investigation.

Melanoidin extract from aged vinegar and pomegranate extract (PE) have recently been shown to mediate mitophagy in injury-induced hepatocytes and cardiomyocytes by increasing Beclin-1 levels [29]. Beclin-1 is a component of the class III PI3K complex important for autophagosome membrane biogenesis [122] and is recently shown to influence mitophagy in cardiac I/R injury via novel regulation of mTORC1 [123]. A role of Beclin-1 in regulating autophagic mitochondrial clearance is further affirmed by another study showing that calpain-2-mediated degradation of Beclin-1 impaired mitophagy in rat hepatocytes [124]. Taken together, the Beclin-1-mTORC1 signalling axis potentially represents a novel signalling route to activate mitophagy and may underscore melanoid-mediated mitophagy. However, it remains unclear if TFEB participates in Beclin-1-mTORC1-regulated mitophagy.

Urolithin A (UA), another metabolite derived from PE, has recently shown to induce mitophagy in *C. elegans* and rodents [15]. UA is one of the hydrolysed end products of ellagitannins found highly enriched in PE. UA supplementation prolongs lifespan in *C. elegans* and consistently increases healthspan in aged worms and mice by preventing age-related muscle deterioration. In *C. elegans*, exposure to UA increased the expression of autophagy (*bec-1*, *sqst-1*, and *vps-34*) and mitophagy (*pink1*, *dct-1*, and *skn-1*) genes that contributed to mitophagy induction. A reduced expression of mitochondrial fusion factors was also observed that aligned with an increase in mitochondrial fragmentation observed in UA-treated cells and tissues. This alteration in mitochondrial dynamics potentially favours efficient autophagic targeting of the fragmented mitochondria to enhance mitophagy flux. The mechanism underlying UA-induced transcriptional upregulation of mitophagy was not explored in the study. It will be interesting to explore if UA potentiates mitophagy via influencing FOXO3 and TFEB, the two major transcriptional activators of mitophagy.

### 4.3. The Hormetic Effect: Polyphenols as Pro-Oxidants to Activate Lysosomal Ca^2+^ Signalling for Mitophagy?

ROS are widely accepted to be damaging molecules. However, emerging evidence suggests ROS also serve important functional roles by acting as signalling molecules to regulate important physiological processes [125, 126]. In the concept of “hormesis,” low doses of ROS stress may be protective by activating stress response pathways that promote longevity [127, 128]. Hormesis describes the upregulation of pre-emptive adaptive responses to enhance the readiness of the cells to counteract the onset of more aggressive cellular stress thereby increasing cell resilience [129]. Mitophagy is also subjected to ROS regulation via mitochondrial ROS-mediated lysosome Ca^2+^ signalling pathway. Low level mitochondrial ROS therefore may facilitate mitochondrial hormesis by priming mitophagy on standby to attenuate oxidative stress through efficient removal of dysfunctional mitochondria to protect mitochondrial and cellular redox homeostasis.

Interestingly, polyphenols also elicit hormetic effects via its pro-oxidant properties when administered at regulated doses. The pro-oxidant effects often involve interactions of polyphenols with transition metal ions [130]. An example is curcumin which exhibits a pro-oxidant property at very low doses (≤1 *μ*M) in the presence of Cu(II) but operates primarily as an autophagy inducer when present in the range of 5–10 *μ*M, wherein it mediates the protective effects of autophagy [131]. How do pro-oxidant effects mediated by polyphenols benefit cells? It is tempting to postulate that a plausible mechanism underlying hormetic effects of polyphenol pro-oxidant properties is the induction of mitophagy via ROS-lysosomal Ca^2+^ signalling. In this model, we propose that low levels of ROS generated by polyphenols when administered in an acute or nonlethal dose may stimulate lysosome-Ca^2+^ signalling to activate TFEB to increase transcription of autophagy-lysosomal and mitophagy genes (Figure 2(a)). Under physiological conditions, expression of these genes may increase autophagy-lysosomal fitness to prime the mitochondria for efficient transit to mitophagy in the event of mitochondrial stress.

## 5. Concluding Thoughts

In recent years, transcriptional modulation of autophagy has become a focus of attention owing to the identification of TFEB. TFEB activation has also been recently shown to regulate mitophagy. The identification of ROS-lysosome-Ca^2+^ signalling to activate TFEB presents an exciting interface for crosstalk between mitochondria and the lysosome to modulate mitochondria quality control. Interestingly, impairment in the activity of one organelle affects the other. For example, in Pompe disease, a lysosomal storage disorder, the impaired lysosome function is associated with perturbed mitochondrial membrane potential and Ca^2+^ homeostasis [132]. Mitochondrial dysfunction similarly leads to accumulation of damaged lysosomes in mouse fibroblast cells deficient in mitophagy factors [133]. For the latter, accumulation of ROS is the cause for lysosomal impairment, which further highlights the importance of lysosome as a ROS sensing hub to upregulate mitophagy (via TFEB) to remove damaged mitochondria and restore lysosome integrity.

Most studies thus far have only examined the role of polyphenols in general autophagy modulation. Very few polyphenols have been identified to specifically regulate mitophagy, and even lesser is known about polyphenols that regulate mitophagy via transcriptional control. Nonetheless, the identification of several phenolic compounds that could influence mitochondrial clearance via FOXO3a and TFEB signalling highlights the potential of dietary intake as an avenue for mitophagy upregulation in humans. It will be exciting to explore the prospect of augmenting mitophagy through polyphenol consumption as a therapeutic approach towards mitochondria-related diseases.
